# Synthesis of Bistetrahydroquinolines as Potential Anticholinesterasic Agents by Double Diels-Alder Reactions 

**DOI:** 10.3390/molecules181012951

**Published:** 2013-10-17

**Authors:** Yorley Duarte, Margarita Gutiérrez, Luis Astudillo, Jans Alzate-Morales, Natalia Valdés

**Affiliations:** 1 Laboratorio Síntesis Orgánica, Instituto de Química de Recursos Naturales, Universidad de Talca, Casilla 747, Talca 3460000, Chile; E-Mails: yduarte@utalca.cl (Y.D.); lastudi@utalca.cl (L.A.); 2 Centro de Bioinformática y Simulación Molecular, Universidad de Talca, 2 Norte 685, Casilla 721, Talca 3460000, Chile; E-Mails: jalzate@utalca.cl (J.A.-M.); nvaldesp@alumnos.utalca.cl (N.V.)

**Keywords:** bistetrahydroquinolines, Diels-Alder reaction, AChE and BuChE inhibitors, molecular docking, MM-GBSA

## Abstract

The tetrahydroquinoline ring system is a unit found in many biologically active natural products and pharmacologically relevant therapeutic agents. A new series of bistetrahydroquinolines (*bis-*THQs) was synthesized using imino Diels-Alder reactions between dialdehydes, anilines and *N*-vinyl-2-pyrrolidone (NVP). The notable features of this procedure are mild reaction conditions, greater selectivity and good yields of products. In addition, the inhibitory activity against acetylcholinesterase (AChE) and butyrylcholinesterase (BuChE) of some selected derivatives is reported. The feasible binding modes of these active compounds, within AChE and BuChE binding sites, were predicted by molecular docking experiments and their binding affinity was estimated by means of free energy calculations through the MM-GBSA approximation.

## 1. Introduction

Heterocyclic compounds, especially nitrogen heterocycles, are the most important class of compounds in the pharmaceutical and agrochemical industries [1]. The tetrahydroquinoline (THQ) system, constitutes a privileged substructure found in numerous biologically active natural products and pharmacologically relevant therapeutic agents [2].

The THQ nucleus has been found to possess a wide range of biological activities, including psychotropic activity [3], anti-allergenic [4], antitumoral [5], antimalarial [6], anti-bacterial [7], antifungal [8,9], cardiovascular activity [10], antiviral activity [11,12], *etc*. Also, these kind of molecules can act as γ-secretase inhibitors for the treatment of Alzheimer’s disease [13], as platelet aggregation inhibitors [14] and modulators of HIV transcription [15].

Among those, Alzheimer’s disease (AD), characterized by progressive cognitive impairment, has been raising much interest as the most common cause of dementia in elderly people. It is also a multifactorial disorder involving the malfunction of different biochemical pathways in which certain enzymes play a key role [16]. Among these enzymes are cholinesterases, which have become important therapeutic targets for AD treatments. For example, AChE catalyzes the hydrolysis of acetylcholine, decreasing its availability in the synaptic space [17], and inhibitors of AChE have been used as palliative drugs for AD, including synthetic compounds as tacrine [18], donepezil [19], galanthamine [20] and rivastigmine [21], which have all been proven to improve a little the situation of AD patients. However, it is important to know that the use of different biological entities that are involved in the same pathology (AChE and BuChE), it is widely accepted and can be a better strategy to block the course of multifactorial diseases rather than just reducing their symptoms [22]. Therefore, and taking into account the effectiveness of this methodology for the treatment of AD, it is necessary to focus our research in obtaining better AChE and BuChE inhibitors.

Due to their broad biological activity, as mentioned in the background, THQ compounds have been considered a good starting material in the search of novel inhibitors of the enzymes AChE and BuChE.

Many synthetic routes to THQs are known, but due to the importance associated with their biological activity, the development of new synthetic approaches remains an active research area [23]. The imino Diels-Alder reaction between aldimines and electron-rich alkenes is probably the most powerful synthetic tool for the construction of this kind of heterocyclic compound [24,25]. This reaction has been extensively studied with protic acids; different Lewis [26] and Brönsted acids [27] and also lanthanide triflates [28] have been used as efficient catalysts for the synthesis of THQs. This method allows the generation of THQs derivatives with a high degree of structural diversification [29].

The main purposes of our work were: first, to develop a simple and efficient synthesis protocol for *bis-*THQ derivatives with several degrees of structural diversity; second, to study their biological activity as inhibitors of the enzymes AChE and BuChE and third, to explain their binding modes of interaction with those molecular targets, aiming in particular at estimating the binding free energy of the compounds.

## 2. Results and Discussion

### 2.1. Chemistry

**Scheme 1:**
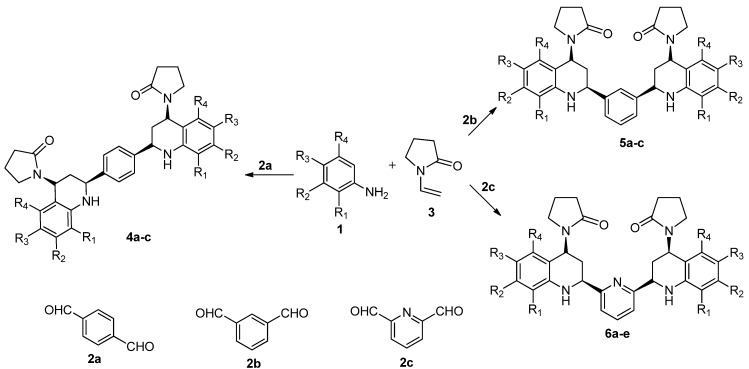
Synthesis of novel series of *bis-*THQs, through the r.t. Povarov reaction promoted by BiCl_3_ and MeCN.

We report here the synthesis of a new series of 1,4-bis(heteroaryl-4'-(2-oxopirrolidinyl-1)-1',2',3',4'-tetrahydroquinolin-2-yl)benzenes **4a**–**c**, 1,3-bis(heteroaryl-4'-(2-oxopirrolidinyl-1)-1',2',3',4'-tetrahydroquinolin-2-yl)benzenes **5a**–**c**, and 2,6-bis(heteroaryl-4'-(2-oxopirrolidinyl-1)-1',2',3',4'-tetrahydroquinolin-2-yl)pyridine derivatives **6a**–**e**. They were prepared by imino Diels-Alder cycloaddition between different substituted anilines **1**, dialdehydes (terephthalaldehyde (**2a**), isophthalaldehyde (**2b**), 2,6-pyridinedicarboxaldehyde **2(c**)), and NVP **3** as alkene, using acetonitrile as solvent in the presence of 20 mol% of bismuth trichloride (III) as catalyst (Scheme 1). It must be taken into consideration that bismuth compounds have attracted attention due to their low toxicity, low cost, water tolerant catalyst [30] and good stability in several reactions such as the imino Diels-Alder (Povarov) reaction.

The reactions proceeded efficiently at room temperature under mild conditions to give the *bis-*THQ products as stable solids in 22%–67% yields after purification using SiO_2_ column chromatography, depending of the structural variations in aromatic anilines and dialdehydes (Table 1).

**Table 1:** Reaction times and yields of *bis-*THQs **4a**‒**c**, **5a**–**c** and **6a**–**e**.

Compound	R1	R2	R3	R4	Mp (°C)	Yield (%)	Reaction Time (h)
4a	H	H	H	H	250–257	60	7
4b	H	H	CH_3_	H	295–298	75	7
4c	H	H	O-CH_3_	H	218–221	22	5
5a	H	H	H	H	221–225	67	7
5b	H	H	CH_3_	H	190–195	51	8
5c	H	H	O-CH_3_	H	237–239	58	6
6a	H	H	H	H	155–158	46	7
6b	H	H	CH_3_	H	187–190	72	5
6c	H	H	O-CH_3_	H	173–178	34	5
6d	H	H	NO_2_	H	202–206	65	10
6e	H	CH_3_	H	CH_3_	157–161	25	9

In some cases, the reaction provides the corresponding products depending on the substituents of the aromatic ring of aniline. In anilines bearing electron-donating groups, it was found that the reaction proceeded faster than the ones bearing electron-withdrawing groups (Table 1). This observation could be attributed to the higher nucleophilic activity of aromatic amines with electron-donating groups.

The products were obtained predominantly as *cis*-diasteroisomers; this configuration was determined by ^1^H-NMR spectroscopy and assigned on the basis of the corresponding coupling constants. The stereochemistry was also confirmed by 1D selective NOE NMR experiments. All ^1^H-NMR spectra of the synthesized *bis*-THQs were very similar and characterized by the presence of three groups of signals at δ 8.0–6.5, indicating the presence of aromatic protons. Similarly, we observed the tetrahydoquinolinic H-2', H-2'' and H-4', H-4'' proton signals at δ 6.0–4.5 and with large coupling constant values (between 8.5–12.0 Hz for H-4' and H-2', respectively). Finally, the δ 2.9–1.5 zone showed multiplets corresponding to the proton signals of the pyrrolidone group. NOE experiments support the relative *trans*-orientation of H-2', H-3' and H-4'. Thus, in the case of **4b**, **5b** and **6c** the selective irradiation of H-2' produced an NOE effect over H-3' and H-4', for the three compounds.

Notably, the high value of the coupling constants found in the proton signals corresponding to H-2', H-2'', H-4' and H-4'' confirmed the *trans* configuration. Therefore, and, based on these results, we can conclude that the compounds obtained correspond to the *endo* cycloadducts of the imino Diels-Alder reaction. Besides, by a comparison with previous reports [31,32,33] for analogous THQs, we assumed that the major isomer has a *cis*-configuration of the C-2 and C-4 substituents.

**Scheme 2:**
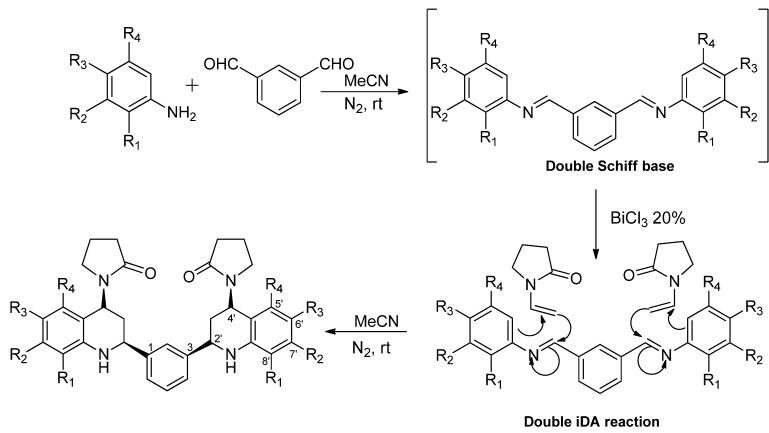
Plausible mechanism reaction in the synthesis of the *bis-*THQs.

From these results, we propose the following possible mechanism to explain the product formation (Scheme 2). This procedure allows a cycloaddition reaction between substituted anilines **1** and dialdehydes **2**, generating a double Schiff base, which is activated by the presence of BiCl_3_ as Lewis acid, and by double imino Diels-Alder (iDA) cycloaddition with VNP leads to the formation of the *bis-*THQs.

### 2.2. Biological Activities

The *bis-*THQs **4a**–**c**, **5a**–**c** and **6a**–**e** were tested for inhibition of the enzymes AChE and BuChE, and the inhibitory activity of the newly synthesized compounds was studied using the method described by Ellman [34] to determine the rate of hydrolysis of acetylthiocholine/butyryltiocholine in the presence of the inhibitor. The activity was assayed in comparison with galanthamine as a reference compound. The main reason to use galanthamine was because it belongs to a group of natural products corresponding to the alkaloid family [35], which structurally resembles the compounds that were reported in this work. Galanthamine was also used taking into account previous theoretical studies [36], where it is described as the compound with the major number of interactions and low free energy of binding against AChE. The compound’s selectivity was also tested by determining their inhibitory activity against BuChE, this with the aim to compare both enzymes with the same inhibitors.

BuChE, also called nonspecific cholinesterase or pseudocholinesterase, is able to act on hydrophilic and hydrophobic choline esters. Even though BuChE is closely related to AChE, owing to a larger active site gorge of BuChE, a broader variety of substrates and inhibitors are accepted in its binding site, compared with AChE [37].

Among the 11 compounds synthesized, only two compounds, **5c** and **4c**, showed some biological activity against AChE and BuChE, respectively, with IC_50_ values of 122 µM for **5c** in AChE and 323 µM for **4c** in BuChE. However, these IC_50_ values were not comparable with the standard inhibitor galanthamine (0.54 µM). The other synthesized compounds showed IC_50_ values higher than 500 µg/mL, therefore, they were not interesting as feasible AChE/BuChE inhibitors.

The more active compounds contain a [6-methoxy-1,2,3,4-tetrahydroquinoline] substituent. The presence of other substituents such as 6-methyl or hydrogen decreased the AChE and BuChE inhibitory activity. Furthermore, the large size of the molecules hindered them to accommodate along the gorge of AChE, which is smaller than the BuChE [38]. Interestingly, the compound **5c** has a better selectivity for AChE over BuChE (2.64), perhaps due to the fact this molecule allows greater flexibility in the pocket of this enzyme, as opposed to the structure **4c** that does appear to have a larger size.

### 2.3. Molecular Docking and Binding Affinity Calculations

**Figure 1:**
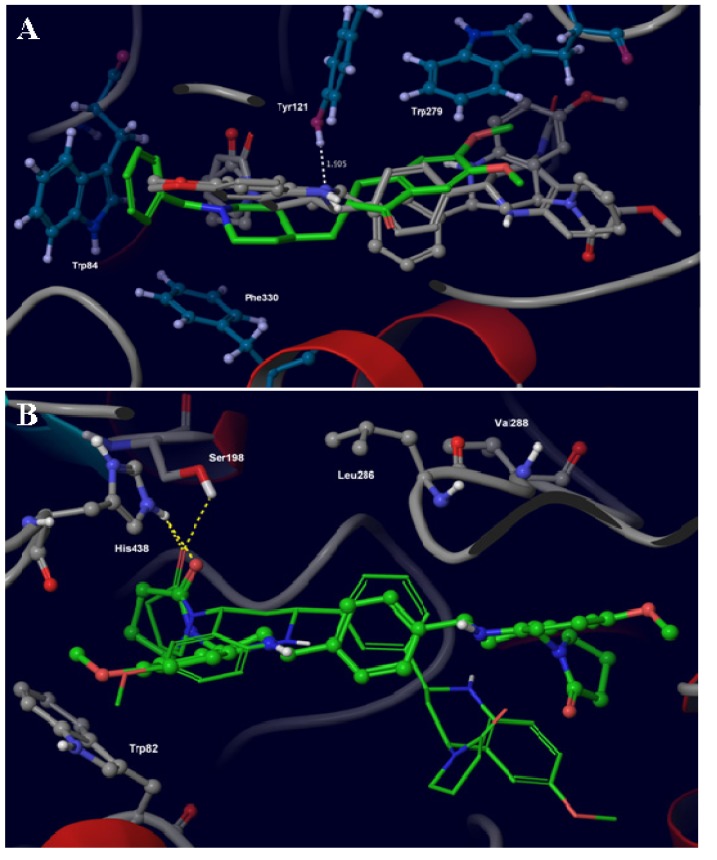
(**A**) Main molecular interactions established by compounds **E2020** (green carbon atoms), **5c** (gray carbon atoms and ball-stick representation) and **4c** (gray carbon atoms and stick representation) within AChE binding site. (**B**) Main molecular interactions established by compounds **5c** (green carbon atoms and thin stick representation) and **4c** (green carbon atoms and width stick representation) within BuChE binding site. Only main amino acids within AChE and BuChE are sketched for the sake of clarity.

Considering the presented *in vitro* results, and in order to obtain a theoretical model for potential binding modes and affinity strength of the most active compounds within the AChE and BuChE binding sites, molecular docking and Molecular Mechanics-Generalized Born Surface Area (MM-GBSA) calculations were performed for compounds **5c** and **4c** against the studied molecular targets. A graphical inspection of the molecular docking results was made in order to explain the possible molecular interactions for the two most active AChE and BuChE inhibitors **5c** and **4c** (See Figure 1).

In Figure 1A is shown an overlay of the structures for compounds **5c**, **4c** and **E2020** that is a member of a large family of N-benzylpiperidine-based AChE inhibitors that were developed, synthesized and evaluated by the Eisai Company in Japan [39], on the basis of QSAR studies [40,41]. The compound **E2020** (green carbon atoms) establishes aromatic stacking interactions against the indole ring of residues Trp84 and Trp279. On the other hand, the charged nitrogen of the piperidine ring makes a cation–π interaction with the phenyl ring of Phe330 [42,43]. The nitrogen atom of the piperidine ring makes a hydrogen bond (H-bond) interaction with residue Tyr121, which is mediated by a water molecule. Finally **E2020** does not interact with the catalytic triad [19].

According to our molecular docking results, and comparing the interactions against model compounds **E2020**, the compounds **5c** and **4c** could establish some of the above-mentioned interactions within the AChE binding site. For instance, both compounds established CH-π interactions with indole ring of Trp84 through the methoxy groups attached to one of the tetrahydroquinoline ring of tested compounds. An aromatic stacking interaction is formed between tetrahydroquinoline ring of compound **5c** and the indole ring of residue Trp279. The 1,4 substitution pattern of tetrahydroquinoline rings around the central phenylene group makes this interaction to be broken for compound **4c**. On the other hand both compounds established a hydrogen bond interaction between nitrogen atom in tetrahydroquinoline ring and hydroxyl group at residue Tyr121. None of the compounds formed any interactions with catalytic triad.

The molecular interactions, derived from docking experiments, for compounds **5c** and **4c** within the BuChE active site are shown in Figure 1B. As can be seen, the binding site pocket in BuChE seems to be less crowded that the corresponding one in AChE, which is in agreement with previous work that reported that smaller residues like Leu286 and Val288, instead of Phe295 and Phe297 in AChE, are responsible for this [35].

The only molecular interactions present in our models suggest that compound **5c** makes multiple H-bond contacts, through the oxygen atom at pyrrolidone ring, with two residues in the catalytic triad in the BuChE active site, namely His438 and Ser198. On the other hand, compound **4c** can only establish H-bond interactions with residue His438. Both compounds seem to be in close contact with residue Trp82 establishing some CH-π interactions through their methoxy groups on the tetrahydroquinoline ring. The remaining molecular structure orientation of compounds within the binding site are quite different due to the substitution patterns obtained for the tetrahydroquinoline rings around the central phenylene scaffold.

Regarding the binding affinity energies obtained from molecular docking and MM-GBSA approximations, and their relationship with molecular interactions discussed above; it is important to clarify that our aim here was to get a rough estimation of the energetic differences observed in binding affinity, and not to obtain a more detailed structure activity relationships for compounds studied. This is supposed to provide us with fresh ideas, mainly from the structural point of view, to redirect the synthesis of new potent and selective THQ derivatives active against AChE/BuChE.

In Table 2 the computational binding affinity energies obtained for compounds **5c** and **4c** in their complexes with the proteins AChE and BuChE can be seen. The docking score for compound **5c** suggest a more favorable interaction within the AChE binding sites than in the BuChE one (−8.63 and −7.98 kcal mol^−1^, respectively), when compared with compound **4c**, with score energies of −7.37 and −8.82 kcal mol^−1^, respectively. This roughly indicates that compound **5c** could interact more strongly with AChE than with BuChE, and the opposite is true for compound **4c**. This is in agreement with the experimental affinity data reported in this work. However a work of caution is important here because the lack of more experimental affinity data to compare with computational findings (*i.e*., only two compounds showed defined IC_50_ values), and due to the molecular size of the studied compounds that should prove to be difficult to accommodate in the AChE binding site. Other important point from our experimental findings is the solubility problems found with the majority of synthesized compounds. In order to get a more precise binding affinity prediction for protein-ligand complexes studied in this work, we performed MM-GBSA calculations.

**Table 2:** Protein-ligand binding affinities estimated through molecular docking and MM-GBSA approaches.

	Glide docking score (kcal mol^−1^)		Binding Free Energy (kcal mol^−1^) ^a^	
Target/Ligand	AChE	BuChE	AChE	BuChE
5c	−8.63	−7.98	−30.64	−33.94
4c	−7.37	−8.82	−28.72	−29.27

^a^ Free binding energies were obtained through MM-GBSA approach implemented in Prime module of Schrödinger suite.

This technique is supposed to describe in a more accurate way the solvation events occurring in protein-ligand binding processes. According to those results, compound **5c** binds more strongly to AChE than compound **4c** does (−30.64 and −28.72 kcal mol^−1^, respectively), and regarding the binding of those compounds to BuChE, compound **5c** also binds more strongly than compound **4c** (−33.94 and −29.27 kcal mol^−1^, respectively). The latter free binding energy results are not in agreement with either previous docking results or with experimental binding affinity results, but could help us to understand the molecular determinants for the interaction of these new compounds within the binding sites of the enzymes AChE and BuChE.

Summarizing, molecular docking and MM-GBSA experiments could be interesting approaches to try to predict the activity for this class of *bis-*THQs in AChE and BuChE receptors. Moreover, the different binding patterns for the best inhibitors proposed in this work, could help us to better understand the modes of interaction of new series of *bis-*THQs inhibitors synthesized in our laboratory.

## 3. Experimental

### 3.1. General

Melting points were determined on a Buchi apparatus and are uncorrected. The compounds’ purity was checked by thin layer chromatography (TLC) on silica gel and they were purified by column chromatography. Chemicals were used without further purification. FT-IR spectra were recorded in potassium bromide pellets using a Thermo Nicolet NEXUS 670 FT-IR spectrophotometer, with 0.125 cm^−1^ spectral resolution. ^1^H-NMR (400 MHz) and ^13^C-NMR spectra (100 MHz) were recorded in CDCl_3_ or DMSO-*d_6_*, using a Bruker AM-400 instrument. Chemical shifts are expressed as values relative to TMS as internal standard. High-resolution mass spectrometry ESI-MS and ESI-MS/MS analyses were conducted in a high-resolution hybrid quadrupole (Q) and orthogonal time-of-flight (TOF) mass spectrometer (Waters/Micromass Q-TOF micro, Manchester, UK) with a constant nebuliser temperature of 100 °C. The experiments were carried out in positive ion mode, and the cone and extractor potentials were set at 10 and 3.0 V, respectively, with a scan range of *m*/*z* 150–600. MS/MS experiments were carried out by mass selection of a specific ion in Q1, which was then submitted to collision-induced dissociation (CID) with helium in the collision chamber. The product ion MS analysis was accomplished with the high-resolution orthogonal TOF analyzer. The samples were directly infused into the ESI source, via a syringe pump, at flow rates of 5 µL min^−1^, via the instrument’s injection valve.

*General procedure for the preparation of 1,4-bis(heteroaryl-4'-(2-oxopirrolidinyl-1)-1',2',3',4'-tetra-hydroquinolin-2-yl)benzenes* **4a**–**c**, *1,3-bis(heteroaryl-4'-(2-oxopirrolidinyl-1)-1',2',3',4'-tetrahydro-quinolin-2-yl)benzenes* **5a**–**c**, *and 2,6-bis(heteroaryl-4'-(2-oxopirrolidinyl-1)-1',2',3',4'-tetrahydro-quinolin-2-yl)pyridines* **6a**–**e**. A mixture of *p*-anisidine (3 mmol) and isophthalaldehyde (1.5 mmol) in anhydrous CH_3_CN (5 mL) was stirred at room temperature under N_2_ for 1 h. BiCl_3_ (20 mol%) was added. Over a period of 20 min, a solution the NVP (3.1 mmol) in CH_3_CN (5 mL) was added dropwise. The resulting mixture was stirred for 8–10 h. After completion of the reaction as indicated by TLC, the reaction mixture was diluted with (15 mL) and extracted with ethyl acetate (3 × 10 mL). The organic layer was separated, and dried with Na_2_SO_4_. The organic solvent was removed *in vacuo* and the resulting product was purified by column chromatography (silica gel, petroleum ether/ethyl-acetate) to afford the pure *bis*-THQs.

*1,4-bis(4'-(2-Oxopyrrolidinyl-1)-1',2',3',4'-tetrahydroquinolin-2-yl)benzene* (**4a**). White solid; Yield 60%; Mp 250–257 °C; IR (cm^−1^): 3315, 2920, 1659, 1486, 1282. ^1^H-NMR (CDCl_3_, DMSO-*d_6_*) δ ppm: 7.45 (4H, s, 2-H, 3-H, 5-H and 6-H), 7.08 (2H, t, *J* *=* 7.8 Hz, 7'-H and 7''-H), 6.88 (2H, d, *J* *=* 8.0 Hz, 5'-H and 5''-H), 6.73 (2H, t, *J* *=* 7.8 Hz, 6'-H and 6''-H), 6.60 (2H, d, *J* *=* 8.0 Hz, 8'-H and 8''-H), 5.74 (2H, t, *J* *=* 9.5 Hz 4'-H and 4''-H), 4.63 (2H, m, 2'-H and 2''-H), 3.99 (2H, s, N-H), 3.28–3.19 (4H, m, 5-H_pyrr_ and 5'-H_pyrr_), 2.59–2.43 (4H, m, 3-H_pyrr_ and 3'-H_pyrr_), 2.13–2.10 (4H, m, 3'-H and 3''-H), 2.05–1.99 (4H, m, 4-H_pyrr_ and 4'-H_pyrr_). ^13^C-NMR (CDCl_3_, DMSO-*d_6_): * 18.2 (2), 31.4 (2), 42.30 (2), 48.4 (4), 56.1 (2), 114.9 (2), 118.3 (2), 118.8 (2), 126.9 (4), 128.2 (4), 142.8 (2), 145.8 (2), 175.85 (2). MS (ESI, *m*/*z*): 507.41 ([M+H])^+^, 529.34 ([M+Na])^+^.

*1,4-bis(6'-Methyl-4'-(2-oxopyrrolidinyl-1)-1',2',3',4'-tetrahydroquinolin-2-yl)benzene* (**4b**). White solid; Yield 75%; Mp 295–298 °C; IR (cm^−1^): 3318, 2920, 1661, 1496, 1281. ^1^H-NMR (CDCl_3_) δ ppm: 7.44 (4H, s, 2-H, 3-H, 5-H and 6-H), 6.88 (2H, d, *J* *=* 8.0 Hz, 7'-H and 7''-H), 6.69 (2H, s, 5'-H and 5''-H), 6.52 (2H, d, *J* *=* 8.0 Hz, 8’-H and 8''-H), 5.71 (2H, dd, *J* *=* 9.6, 8.4 Hz, 4'-H and 4''-H), 4.59–4.56 (2H, m, 2'-H and 2''-H), 3.86 (2H, s, N-H), 3.26−3.21 (4H, m, 5-H_pyrr_ and 5'-H_pyrr_), 2.56–2.45 (4H, m, 3-H_pyrr_ and 3'-H_pyrr_), 2.23 (6H, s, 6'-CH_3_), 2.11–2.09 (4H, m, 3'-H and 3''-H), 2.07–2.01 (4H, m, 4-H_pyrr_ and 4'-H_pyrr_). ^13^C-NMR (CDCl_3_, DMSO-*d_6_*) δ: 18.6 (2), 20.9 (2), 31.4 (2), 42.30 (2), 48.4 (4), 56.1 (2), 114.9 (2), 118.3 (2), 118.8 (2), 126.9 (4), 128.2 (4), 142.8 (2), 145.8 (2), 175.85 (2). MS (ESI, *m*/*z*): 535.67 ([M+H])^+^, 557.68 ([M+Na])^+^.

*1,4-bis(6'-Methoxy-4'-(2-oxopyrrolidinyl-1)-1',2',3',4'-tetrahydroquinolin-2-yl)benzene* (**4c**). Crystalline solid; Yield 22%; Mp 218–221 °C; IR (cm^−1^): 3378, 2956, 1659, 1497, 1284. ^1^H-NMR (CDCl_3_, DMSO-*d_6_*) δ ppm: 7.99 (4H, s, 2-H, 3-H, 5-H and 6-H), 6.71 (2H, d, *J* *=* 2.4 Hz, 5'-H and 5''-H), 6.60 (2H, d, *J* *=* 8.8 Hz, 7'-H and 7''-H), 6.50 (2H, d, *J* *=* 8.6 Hz, 8'-H and 8''-H), 5.74 (2H, dd, *J* *=* 11.2, 10.8 Hz, 4'-H and 4''-H), 4.64–4.61 (2H, m, 2'-H and 2''-H), 3.90 (2H, s, N-H), 3.74 (6H, s, O-CH_3_), 3.29–3.23 (4H, m, 5-H_pyrr_ and 5'-H_pyrr_), 2.55–2.46 (4H, m, 3-H_pyrr_ and 3'-H_pyrr_), 2.16–2.14 (4H, m, 3’-H and 3''-H), 2.13–2.01 (4H, m, 4-H_pyrr_ and 4'-H_pyrr_). MS (ESI, *m*/*z*): 567.72 ([M+H])^+^, 589.71 ([M+Na])^+^.

*1,3-bis(4'-(2-Oxopyrrolidinyl-1)-1',2',3',4'-tetrahydroquinolin-2-yl)benzene* (**5a**). Green solid; Yield 67%; Mp 221–225 °C; IR (cm^−1^): 3319, 2943, 1670, 1484, 1250 cm^−1^. ^1^H-NMR (CDCl_3_, DMSO-*d_6_*) δ ppm: 7.52 (1H, s, *2*-H), 7.37 (3H, m, 4-H and 6-H and 5-H), 7.07 (2H, t, *J* *=* 7.8 Hz, 7'-H and 7''-H), 6.88 (2H, m, 5'-H and 5''-H), 6.73 (2H, m, 6'-H and 6''-H), 6.61 (2H, m, 8'-H, 8''-H), 5.72 (2H, m, 4'-H and 4''-H), 5.30 (2H, s, N-H), 4.62 (2H, 2'-H and 2''-H), 3.23–3.25 (4H, m, 5-H_pyrr_ and 5'-H_pyrr_), 2.55–2.43 (4H, m, 3-H_pyrr_ and 3'-H_pyrr_), 2.15–2.03 (8H, m, 3'-H and 3''-H, 4-H_pyrr_ and 4'-H_pyrr_). MS (ESI, *m*/*z*): 507.51 ([M+H])^+^, 529.45 ([M+Na])^+^.

*1,3-bis(6'-Methyl-4'-(2-oxopyrrolidinyl-1)-1',2',3',4'-tetrahydroquinolin-2-yl)benzene* (**5b**). Crystalline solid; Yield 51%; Mp 190–195 °C; IR (cm^−1^): 3315, 2920, 1670, 1511, 1284 cm^−1^. ^1^H-NMR (CDCl_3_) δ ppm: 7.55 (1H, s, *2*-H), 7.37 (3H, s, 4-H and 6-H and 5-H), 6.88 (2H, d, *J* *=* 8.0 Hz, 7'-H and 7''-H), 6.69 (2H, s, 5'-H and 5''-H), 6.54 (2H, d, *J* *=* 8.0 Hz, 8'-H and 8''-H), 5.71 (2H, dd, *J* *=* 10.8, 10.8 Hz, 4'-H and 4''-H), 5.30 (2H, s, N-H), 4.59 (2H, d, *J* *=* 10.4, 10.0 Hz,2'-H and 2''-H), 3.26–3.21 (4H, m, 5-H_pyrr _and 5'-H_pyrr_), 2.60–2.45 (4H, m, 3-H_pyrr _and 3'-H_pyrr_), 2.23 (6H, s, 6'-CH_3_), 2.14–2.10 (4H, m, 3'-H and 3''-H), 2.03–1.99 (4H, m, 4-H_pyrr _and 4'-H_pyrr_). MS (ESI, *m*/*z*): 535.71 ([M+H])^+^, 5557.71 ([M+Na])^+^.

*1,3-bis(6'-Methoxy-4'-(2-oxopyrrolidinyl-1)-1',2',3',4'-tetrahydroquinolin-2-yl)benzene* (**5c**). White solid; Yield 58%; Mp 237–239 °C; IR (cm^−1^): 3325, 2948, 1671, 1509, 1280 cm^−1^. ^1^H-NMR (CDCl_3_) δ ppm: 7.54 (1H, s, *2*-H), 7.38 (3H, m, 4-H and 6-H and 5-H), 6.72 (2H, d, *J* *=* 7.8 Hz, 5'-H and 5''-H), 6.58 (2H, d, *J* *=* 8.8 Hz, 7'-H and 7''-H), 6.50 (2H, s(br), 8'-H and 8''-H), 5.74 (2H, dd, *J* *=* 10.0, 10.0 Hz, 4'-H and 4''-H), 5.32 (2H, s, N-H), 4.55 (2H, dd, *J* *=* 10, 10 Hz, 2'-H and 2''-H), 3.75 (6H, s, O-CH_3_), 3.31–3.22 (4H, m, 5-H_pyrr_ and 5'-H_pyrr_), 2.57–2.45 (4H, m, 3-H_pyrr_ and 3'-H_pyrr_), 2.14–2.11 (4H, m, 3'-H and 3''-H), 2.09–2.01 (4H, m, 4-H_pyrr_ and 4'-H_pyrr_). MS (ESI, *m*/*z*): 567.66 ([M+H])^+^, 589.65 ([M+Na])^+^.

*2,6-bis(4'-(2-Oxopyrrolidinyl-1)-1',2',3',4'-tetrahydroquinolin-2-yl)pyridine* (**6a**). White solid; Yield 46%; Mp 155–158 °C; IR (cm^−1^): 3363, 2918, 1659, 1490, 1286. ^1^H-NMR (CDCl_3_, DMSO-*d_6_*) δ ppm: 7.77 (1H, t, *J* *=* 7.8 Hz, *4*-H), 7.39 (2H, d *J* *=* 7.8 Hz, 3-H and 5-H), 7.10 (2H, t, *J* *=* 7.8 Hz, 7'-H and 7''-H), 6.90 (2H, d, *J* *=* 8.0 Hz, 5'-H and 5''-H), 6.74 (4H, m, 6'-H, 6''-H and 8'-H and 8''-H), 5.80 (2H, m, 4'-H and 4''-H), 5.31 (2H, s, N-H), 4.75 (2H, *m*, 2'-H and 2''-H), 3.22 (4H, m, 5-H_pyrr_ and 5'-H_pyrr_), 2.58–2.45 (4H, m, 3-H_pyrr_ and 3'-H_pyrr_), 2.38–2.32 (4H, m, 3'-H and 3''-H), 2.06–2.00 (4H, m, 4-H_pyrr_ and 4'-H_pyrr_). MS (ESI, *m*/*z*): 508.33 ([M+H])^+^, 530.32 ([M+Na])^+^.

*2,6-bis(6'-Methyl-4'-(2-oxopyrrolidinyl-1)-1',2',3',4'-tetrahydroquinolin-2-yl)pyridine* (**6b**). Black solid; Yield 72%; Mp 187–190 °C; IR (cm^−1^): 3397, 2916, 1655, 1495, 1278. ^1^H-NMR (CDCl_3_) δ ppm: 7.75 (1H, t, *J* *=* 8.0 Hz, 4-H), 7.37 (2H, d, *J* *=* 6.0 Hz, 3-H and 5-H), 6.91 (2H, d, *J* *=* 8.0 Hz, 7'-H and 7''-H), 6.70–6.67 (4H, m, 5'-H and 5''-H, 8'-H and 8''-H), 5.75 (2H, dd, *J* *=* 11.6, 12.4 Hz, 4'-H and 4''-H), 5.33 (2H, s, N-H), 4.70 (2H, m, 2'-H and 2''-H), 3.25–3.18 (4H, m, 5-H_pyrr_ and 5'-H_pyrr_), 2.56–2.48 (4H, m, 3-H_pyrr_ and 3'-H_pyrr_), 2.34–2.30 (4H, m, 3'-H and 3''-H), 2.24 (6H, s, 6'-CH_3_), 2.05–1.99 (4H, m, 4-H_pyrr_ and 4’-H_pyrr_). ^13^C-NMR (CDCl_3_, DMSO-*d_6_*) δ: 17.9 (2), 20.31 (2), 31.1 (2), 42.1 (2), 47.9 (4), 55.9 (2), 115.4 (2), 119.0 (2), 126.7 (2), 128.7 (2), 138.1 (1), 145.0 (2), 159.1 (2), 175.5 (2). MS (ESI, *m*/*z*): 536.85 ([M+H])^+^, 558.85 ([M+Na])^+^.

*2,6-bis(6'-Methoxy-4'-(2-oxopyrrolidinyl-1)-1',2',3',4'-tetrahydroquinolin-2-yl)pyridine* (**6c**). crystalline solid; Yield 34%; Mp 173–178 °C; IR (cm^−1^): 3395, 2916, 1672, 1490, 1277. ^1^H-NMR (CDCl_3_, DMSO-*d_6_*) δ ppm: 7.75 (1H, t, *J* *=* 7.7 Hz, *4*-H), 7.36 (2H, d, *J* *=* 7.8 Hz, 3-H and 5-H), 6.74–6.68 (4H, m, 7'-H and 7''-H, 5'-H and 5''-H), 6.49 (2H, d, *J* *=* 2.1 Hz 8'-H and 8''-H), 5.76 (2H, dd, *J* *=* 11.6, 11.4 Hz, 4'-H and 4''-H), 5.30 (2H, s, N-H), 4.69–4.65 (2H, dd, *J* *=* 11.5, 11.7 Hz, 2'-H and 2''-H), 3.74 (6H, s, O-CH_3_), 3.25–3.20 (4H, m, 5-H_pyrr_ and 5'-H_pyrr_), 2.57–2.48 (4H, m, 3-H_pyrr_ and 3'-H_pyrr_), 2.36–2.32 (4H, m, 3'-H and 3''-H), 2.08–1.98 (4H, m, 4-H_pyrr_ and 4'-H_pyrr_). ^13^C-NMR (CDCl_3_, DMSO-*d_6_*) δ: 17.9 (2), 31.1 (2), 42.1 (2), 48.2 (4), 55.53 (2), 56.2 (2), 111.7 (2), 114.1 (2), 116.7 (2), 119.0 (2), 120.3 (2), 139.1 (2), 152.5 (1), 160.1 (2), 175.5 (2). MS (ESI, *m*/*z*): 568.57 ([M+H])^+^, 590.56 ([M+Na])^+^.

*2,6-bis(6'-Nitro-4'-(2-oxopyrrolidinyl-1)-1',2',3',4'-tetrahydroquinolin-2-yl)pyridine* (**6d**).Brown solid; Yield 65%; Mp 202–206 °C; IR (cm^−1^): 3397, 2916, 1655, 1495, 1278. ^1^H-NMR (CDCl_3_) δ ppm: 8.09 (5H, d, *J* *=* 8,8 Hz, 4-H, 3-H and 5-H, 7'-H and 7''-H), 6.62–6.68 (4H, m, 5'-H and 5''-H, 8'-H and 8''-H), 5.81–5.74 (2H, m, 4'-H and 4''-H), 4.39 (2H, s, N-H), 4.70 (2H, m, 2'-H and 2''-H), 3.38–3.32 (2H, m, 5-H_pirr_), 3.22–3.16(2H, m, 5'-H_pyrr_), 2.49–2.40 (4H, m, 3-H_pyrr_ and 3'-H_pyrr_), 2.05–1.89 (4H, m, 3'-H and 3''-H), 1.51–1.50 (4H, m, 4-H_pyrr_ and 4'-H_pyrr_). MS (ESI, *m*/*z*): 598.35 ([M+H])^+^, 621.33 ([M+Na])^+^.

*2,6-bis(5',7'-Dimethyl-4'-(2-oxopyrrolidinyl-1)-1',2',3',4'-tetrahydroquinolin-2-yl)pyridine* (**6e**). Brown solid; Yield 25%; Mp 157–161 °C; IR (cm^−1^: 3397, 2916, 1655, 1495, 1282. ^1^H-NMR (CDCl_3_) δ ppm: 7.86 (1H, t, *J* = 7.7 Hz, 4-H), 7.47 (2H, d, *J* = 7.6 Hz, 3-H and 5-H**)**, 6.47 (2H, s, 8'-H and 8''-H), 6.43 (2H, s, 6'-H and 6''-H), 5.62–5.58 (2H, m, 4'-H and 4''-H), 4.77 (2H, s, N-H), 4.62 (2H, d, 2'-H and 2''-H), 2.93–2.89 (2H, m, 5-H_pirr_), 2.76–2.73 (2H, m, 5'-H_pyrr_), 2.69–2.65 (4H, m, 3-H_pyrr_ and 3'-H_pyrr_), 2.54 (6H, s, 7'-CH_3_ and 7''-CH_3_), 2.50–2.45 (4H, m, 3'-H and 3''-H), 2.08 (6H, s, 5'-CH_3_ and 5''-CH_3_), 1.77–1.74 (4H, m, 4-H_pyrr_ and 4'-H_pyrr_).

### 3.2. Biological Assays

These activities were evaluated in 96-well plates as described, de la Torre *et al*. [44]. All compounds were evaluated in the range of 500–32 µg/mL.

### 3.3. Computational Details

To obtain information about enzyme inhibitor interactions that might help us to explain the structural requirements for AChE and BuChE activity and selectivity, a docking study was performed. The crystallographic structures of AChE in complex with donepezil inhibitor (pdb code 1EVE) and BuChE in complex with benzoic acid (pdb code 3O9M) were used to dock the derivatives under study.

The former crystallographic structure (1EVE) was selected because it represents the molecular interactions of a well-studied inhibitor compound (**E2020**) within the AChE binding site. The site, the reported data showed that compound **E2020** has high selectivity (compared with BuChE) and high affinity. All previously reported data helped use to rationalize the interactions of the new synthesized compounds againt AChE/BuChE proteins.

The docking simulations were carried out using software Glide operating the standard-precision (SP) mode. A grid box of 25 Å × 25 Å × 25 Å was centered on the center of mass of respective ligands crystallized with AChE and BuChE protein structures. The remaining Glide docking parameters were used as default. The docking poses for each ligand were analyzed by examining both their relative total energy score and their interaction with residues at binding sites. The five more energetically favorable conformations were selected as the best poses.

The Molecular Mechanics/Generalized Born Surface Area (MM/GBSA) approach was used as implemented in Prime (Prime, version 3.1, Schrödinger, LLC, New York, NY, USA, 2012) module from Schrödinger Suite and using default settings. Protein-ligand complexes were obtained from docking experiments as described before.

## 4. Conclusions

We report here the synthesis of a new group of *bis-*THQ derivatives and the evaluation of their biological activity as AChE and BuChE inhibitors. The iDA reaction was used as a key step of a good methodology for the efficient and general synthesis of a selected series of 1,4- or 1,3- or 1,6-bis(heteroaryl-4'-(2-oxopirrolidinyl-1)-1',2',3',4'-tetrahydroquinolin-2-yl)benzenes (pyridines). Among the obtained compounds, only derivatives **5c** and **4c** exhibited a low activity against both enzymes. With the aim to understand the ligand–protein interactions at molecular level and to gain insight of minimal structural requirements for biological activity, molecular docking and MM-GBSA studies were performed for these two compounds.
